# Relativistic Four-Component DFT Calculations of Vibrational Frequencies

**DOI:** 10.1021/acs.jpca.1c07398

**Published:** 2021-11-29

**Authors:** Katarzyna Jakubowska, Magdalena Pecul, Kenneth Ruud

**Affiliations:** † Faculty of Chemistry, University of Warsaw, 02-093 Warsaw, Poland; ‡ Hylleraas Centre for Quantum Molecular Sciences, Department of Chemistry, UiT − The Arctic University of Norway, N-9019 Tromsø, Norway

## Abstract

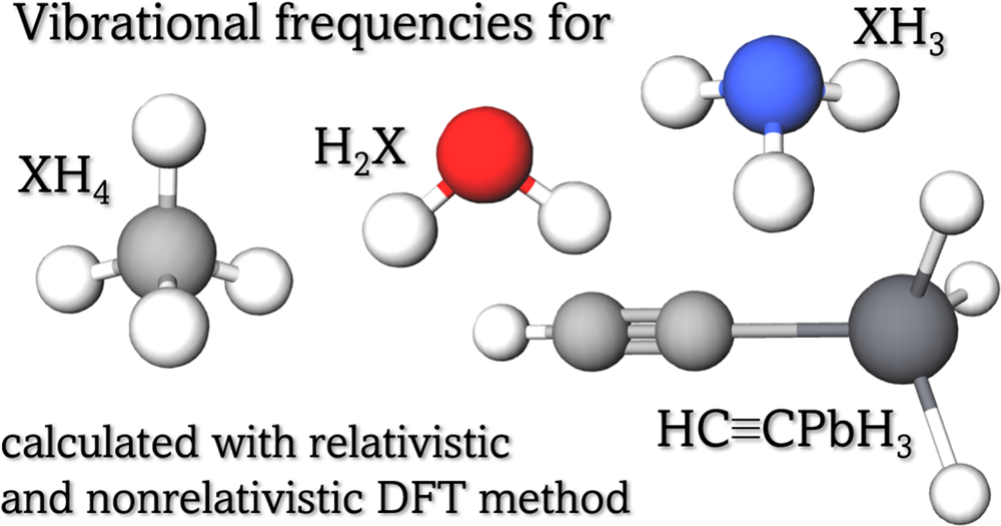

We investigate the effect of relativity on harmonic vibrational frequencies. Density functional theory (DFT) calculations using the four-component Dirac–Coulomb Hamiltonian have been performed for 15 hydrides (H_2_X, X = O, S, Se, Te, Po; XH_3_, X = N, P, As, Sb, Bi; and XH_4_, X = C, Si, Ge, Sn, Pb) as well as for HC≡CPbH_3_. The vibrational frequencies have been calculated using finite differences of the molecular energy with respect to geometrical distortions of the nuclei. The influences of the choice of basis set, exchange–correlation functional, and step length for the numerical differentiation on the calculated harmonic vibrational frequencies have been tested, and the method has been found to be numerically robust. Relativistic effects are noticeable for the heavier congeners H_2_Te and H_2_Po, SbH_3_ and BiH_3_, and SnH_4_ and PbH_4_ and are much more pronounced for the vibrational modes with higher frequencies. Spin–orbit effects constitute a very small fraction of the total relativistic effects, except for H_2_Te and H_2_Po. For HC≡CPbH_3_ we find that only the frequencies of the modes with large contributions from Pb displacements are significantly affected by relativity.

## Introduction

For molecules containing heavy atoms, relativistic effects play a crucial role in their electronic structure and chemical bonding.^1^ Relativistic effects are commonly separated into scalar relativistic effects, which are due to (among other contributions) the mass–velocity and Darwin corrections, and the effects due to the spin–orbit interaction. The former lead for instance to contraction of the inner-shell orbitals (the energies of core levels are lower than those for the nonrelativistic case), and the latter result in the spin–orbit splitting of molecular orbital energy levels. Furthermore, the contraction of the inner-shell orbitals in turn increases the screening of the nuclear charge for the outer-shell electrons, giving rise to an indirect effect that results in expansion of the valence orbitals. These relativistic effects affect the valence orbitals involved with chemical bonding and consequently the potential energy surfaces.^1^

In most cases where potential energy surfaces are concerned, it is sufficient to account for scalar relativistic effects using for example effective core potentials,^2^ but for systems where strong spin–orbit effects may be expected, it is important to have an apparatus to calculate total relativistic effects using the four-component Dirac–Coulomb (or Dirac–Coulomb–Breit) Hamiltonian. Many four-component calculations have been carried out for dissociation energies^1,3^ and molecular gradients (first derivatives of the molecular energy with respect to distortions of the nuclei in the molecule)^4^ as well as equilibrium geometries.^4,5^ In this contribution, we present the results of calculations of the molecular Hessian (second derivatives of the molecular energy with respect to nuclear distortions) and harmonic vibrational frequencies with the Dirac–Coulomb Hamiltonian. Four-component methods for the analytic calculation of molecular Hessians (and thus also harmonic vibrational frequencies) are currently not available in any computational chemistry program package. Instead, our calculations have been carried out with an external driver to the existing program package Dirac.^6^ Test calculations have been performed for hydrides of elements from groups 14 (XH_4_, X = C, Si, Ge, Sn, Pb), 15 (XH_3_, X = N, P, As, Sb, Bi), and 16 (H_2_X, X = O, S, Se, Te, Po) and in addition for the acetylene derivative HC≡CPbH_3_. Vibrational frequencies have been computed with the use of both relativistic and nonrelativistic methods in order to study the importance of the relativistic effects. Such calculations have previously been reported for halogen diatomics^7^ but not for polyatomic molecules.

There are well-established methods of calculating potential energy surfaces, including the molecular Hessian and vibrational frequencies, for molecular systems with substantial relativistic effects through the use of the zeroth-order regular approximation Hamiltonian,^8,9^ other two-component Hamiltonians,^10−13^ and relativistic effective core potentials.^2^ In most cases these are sufficient for rendering the relativistic effects, apart from some systems with very strong spin–orbit coupling, such as some lanthanide compounds.^1,14^ However, a four-component protocol will be useful for benchmarking more approximate treatments of relativistic effects. We have recently also demonstrated that the geometry dependence of NMR spin–spin coupling constants depends more strongly on relativistic effects than the spin–spin coupling constants themselves.^15^ This suggests that relativity may be important for zero-point vibrational (ZPV) corrections to NMR properties. For properties such as spin–spin coupling constants, a full relativistic treatment is necessary, and it is therefore important also to develop tools that allow vibrational frequencies to be calculated at the full four-component level of theory.

## Methods

### Numerical Derivatives

Our program works as an external driver to the Dirac program package.^6^ The method for calculating the molecular Hessian and thus also the harmonic vibrational frequencies is fully numerical. Computation of the Hessian is based on calculating the second derivatives of the molecular energy with respect to geometric distortions of the molecule using simple three-point formulas:^16^
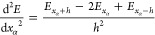
1
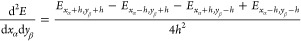
2This means that the Hessian computation involves performing a number of energy calculations in which atoms are displaced from their original positions in all degrees of freedom. In the case of a molecule with *N* atoms, 18*N*^2^ + 1 single-energy computations need to be run to determine the full Hessian. Once the Hessian is obtained, the vibrational frequencies are calculated by diagonalization of the Hessian in its mass-weighted form.

When numerical differentiation is performed, it is important that an appropriate step length (*h* in the above equation) is used to ensure numerically accurate results. We performed test calculations of the vibrational frequencies for the water molecule with a number of different step lengths in the range of 10^–1^–10^–5^ Å. The calculations turned out to be numerically stable for step lengths between 10^–2^–10^–4^ Å. Similar test calculations for the H_2_Po molecule revealed that in the case of heavier atoms, numerical stability shifts in the direction of larger step lengths (5 × 10^–2^ to 5 × 10^–3^ Å). All of the results can be found in the Supporting Information. On this basis, in all of the subsequent calculations we used a step length of 10^–3^ Å. The only exceptions were systems involving the heaviest atoms (Pb, Bi, and Po), for which we used a step length of 10^–2^ Å. Using the differences between vibrational frequencies obtained with step lengths within the range of numerical stability, we were able to estimate the error bars to be about 3 cm^–1^.

In order to test the numerical stability of the three-point formula, additional calculations were carried out using a five-point formula:^16^
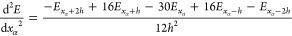
3No significant differences were found in comparison with the three-point formula with the same step length.

Geometry optimizations were performed using the Dirac program^6^ at the same level of theory as for the Hesssian calculations carried out afterward in order to ensure that the molecular gradient was equal to zero (a condition for the harmonic approximation). The convergence treshold for the gradient was 10^–4^.

### Single-Energy Calculations

The four-component Dirac–Kohn–Sham (DKS) energy calculations were carried out with the Dirac program.^6^ The uncontracted aug-cc-pVDZ basis set^17^ on the hydrogen atoms and Dyall’s uncontracted triple-ζ basis set^18−20^ (dyall.v3z) on all of the other atoms were applied together with the B3LYP exchange-correlation functional,^21−24^ unless stated otherwise.

For comparison, four-component calculations with spin–orbit interactions switched off and nonrelativistic calculations were also carried out. In the case of the nonrelativistic computations, the speed of light was scaled to 2000.0 au in the Dirac–Coulomb Hamiltonian, and in the case of the four-component calculations without spin–orbit effects, the calculations were performed with Dyall’s spin-free Hamiltonian^25^ as implemented in Dirac.

Moreover, we carried out an investigation of the dependence of the results on the choice of exchange–correlation functional and basis set. In order to do so, four-component DKS calculations were performed using the PBE0 functional^26^ (to be compared to B3LYP) and also two additional basis sets: (1) the uncontracted aug-cc-pVDZ basis set^17^ on the hydrogen atoms and Dyall’s uncontracted double-ζ basis set (dyall.v2z)^19,20^ on all of the other atoms and (2) the uncontracted aug-cc-pVQZ basis set^17^ on the hydrogen atoms and Dyall’s uncontracted quadrupole-ζ basis set (dyall.v4z)^19,20^ on all of the other atoms.

The convergence threshold for all of the single-energy calculations was 10^–6^.

Since an analytical method for calculating the molecular Hessian is implemented in the Dalton program,^27,28^ some calculations were performed with this program for comparison. We note that Dalton allows only one-component nonrelativistic DFT calculations. All of the Dalton computations were run with the same uncontracted basis set and exchange–correlation functional as above and were carried out using the geometry optimized in Dirac at the nonrelativistic level of theory (the same geometry as the one used for nonrelativistic numerical calculations of vibrational frequencies).

## Results and Discussion

In order to test the newly developed method for calculating harmonic vibrational frequencies, simple systems consisting of three, four, or five atoms have been chosen:

- H_2_X where X = O, S, Se, Te, Po;
- XH_3_ where X = N, P, As, Sb, Bi;
- XH_4_ where X = C, Si, Sn, Pb.

In addition to this, to illustrate the usefulness of the presented method for a larger system, we have calculated vibrational frequencies for an acetylene derivative, HC≡CPbH_3_.

### Influences of the Basis Set and Exchange–Correlation Functional on the Vibrational Frequencies

The results of four-component DFT calculations employing either the B3LYP or PBE0 functional for the vibrational frequencies of H_2_X (X = O, S, Se, Te, Po) can be found in Table 1. In most cases the frequencies obtained with PBE0 are larger, but the differences between the results obtained with PBE0 and B3LYP do not exceed 3% in any case. Taking this into consideration, it seems that in this case B3LYP and PBE0 would produce comparable results. The B3LYP functional has been chosen for the following calculations because of its good performance for vibrational properties reported in the literature.^29−31^

**Table 1:** Vibrational Frequencies for H_2_X: Comparison of Results Calculated with Either the B3LYP or PBE0 Functional^a^

X	functional	ω_1_ [cm^–1^]	ω_2_ [cm^–1^]	ω_3_ [cm^–1^]
O	B3LYP	3918	3815	1623
	PBE0	3983	3877	1630
S	B3LYP	2686	2671	1206
	PBE0	2730	2714	1199
Se	B3LYP	2401	2388	1061
	PBE0	2448	2434	1059
Te	B3LYP	2109	2102	885
	PBE0	2152	2144	885
Po	B3LYP	1846	1829	777
	PBE0	1901	1885	778

a Four-component DKS Hamiltonian with the indicated functional and the aug-cc-pVTZ (on H) + dyall.v3z (on X) basis set.

The results of the four-component DFT calculations of vibrational frequencies for H_2_X systems, carried out with double-ζ, triple-ζ, and quadruple-ζ quality basis sets, can be found in Table 2. The differences between vibrational frequencies obtained with these three basis sets are almost negligible. The biggest differences occur between DZ and QZ for the H_2_O molecule, yet even in this case these differences are not larger than 1% of the values, being at most 31 cm^–1^. In all other cases, the differences do not exceed 10 cm^–1^. In light of the above findings, the triple-ζ-quality basis set appears to be an optimal compromise between accuracy and computational cost, and this basis set has been used in all of the following calculations.

**Table 2:** Vibrational Frequencies for H_2_X: Comparison of Results Calculated with Three Different Basis Sets^a^

X	basis set	ω_1_ [cm^–1^]	ω_2_ [cm^–1^]	ω_3_ [cm^–1^]
O	DZ^b^	3887	3787	1630
	TZ^c^	3918	3815	1623
	QZ^d^	3918	3816	1626
S	DZ^b^	2680	2666	1196
	TZ^c^	2686	2671	1206
	QZ^d^	2688	2675	1208
Se	DZ^b^	2408	2393	1059
	TZ^c^	2401	2388	1061
	QZ^d^	2407	2394	1060
Te	DZ^b^	2114	2107	890
	TZ^c^	2109	2102	885
	QZ^d^	2117	2110	885
Po	DZ^b^	1844	1827	777
	TZ^c^	1846	1829	777
	QZ^d^	1848	1832	778

a Four-component DKS Hamiltonian with the B3LYP functional and the indicated basis set.

b aug-cc-pVDZ (on H) + dyall.v2z (on X).

c aug-cc-pVTZ (on H) + dyall.v3z (on X).

d aug-cc-pVQZ (on H) + dyall.v4z (on X).

### Numerical versus Analytical Hessian

As numerical methods for calculating the molecular Hessian will inevitably have limitations on the numerical accuracy, we have tried to estimate these by comparing the numerical harmonic vibrational frequencies with the results obtained with the analytical nonrelativistic method implemented in the Dalton program. The comparison of the calculated harmonic vibrational frequencies can be found in Table 3. We obtained excellent agreement in case of the H_2_O, H_2_S and H_2_Se molecules.

**Table 3:** Vibrational Frequencies for H_2_X: Comparison of Results Calculated with Relativistic and Nonrelativistic DFT Methods^a^

X	method^b^		ω_1_ [cm^–1^] (A_1_ symmetry, X–H symmetric stretch)	ω_2_ [cm^–1^] (B_2_ symmetry, X–H asymmetric stretch)	ω_3_ [cm^–1^] (A_1_ symmetry, H–X–H bend)
O	num	rel	3918	3815	1623
		no SO	3902	3799	1627
		nrel	3920	3817	1623
	anal	nrel	3920	3818	1623
	experimental^32^		3756	3657	1595
S	num	rel	2686	2671	1206
		no SO	2685	2671	1205
		nrel	2690	2676	1204
	anal	nrel	2690	2676	1205
	experimental^32^		2626	2615	1183
Se	num	rel	2401	2388	1061
		no SO	2404	2390	1061
		nrel	2416	2404	1058
	anal	nrel	2418	2406	1059
	experimental^32^		2358	2345	1034
Te	num	rel	2109	2102	885
		no SO	2122	2115	888
		nrel	2147	2142	884
	anal	nrel	2147	2142	885
	experimental^33^		2072	2065	861
Po	num	rel	1845	1828	775
		no SO	1977	1972	812
		nrel	2032	2031	806
	anal	nrel	2032	2030	809

a B3LYP functional, aug-cc-pVTZ (on H) + dyall.v3z (on X) basis set.

b Fundamental vibrational frequencies are reported for the experimental data. For the calculated results, the following notation is used: num, numerical; anal, analytic; rel, relativistic; nrel, nonrelativistic; no SO, no spin–orbit coupling.

### Influence of Relativity on the Vibrational Frequencies

Harmonic vibrational frequencues calculated with the relativistic and nonrelativistic methods are summarized in Tables 3 –5. As can be noted, in almost all cases the relativistic vibrational frequencies are smaller than the corresponding nonrelativistic values, that is, relativity decreases the bond strength. In the case of the H_2_X systems, relativistic effects are significant for H_2_Te (2% for ω_1_ and ω_2_) and H_2_Po (10% for ω_1_ and ω_2_, 3% for ω_3_). Also in the case of the XH_3_ molecules, relativistic effects are not negligible for the two heaviest congeners, 2% for ω_1_ and ω_2_ in SbH_3_ and 8% for ω_1_, 5% for ω_2_, and 2% for ω_3_ in BiH_3_. In the case of the XH_4_ molecules, relativistic effects are negligible for all but one mode, ω_3_ (E symmetry mode), for SnH_4_ (2%) and PbH_4_ (6%). It should be stressed here that the precentage change in the values when calculated with relativistic and nonrelativistic methods varies for each vibrational mode.

**Table 4:** Vibrational Frequencies for XH_3_: Comparison of Results Calculated with Relativistic and Nonrelativistic DFT Methods^a^

X	method^c^	ω_1_ [cm^–1^] A_1_ symmetry, X–H symmetric stretch	ω_2_ [cm^–1^] (E symmetry, X–H asymmetric stretch)^b^	ω_3_ [cm^–1^] (E symmetry, H–X–H scissor)^b^	ω_4_ [cm^–1^] (A_1_ symmetry, X–H wag)
N	rel	3596	3476	1661	1019
	no SO	3584	3465	1663	1029
	nrel	3587	3467	1662	1024
	experimental^32^	3444	3337	1627	950
P	rel	2385	2374	1136	1016
	no SO	2385	2375	1136	1016
	nrel	2389	2379	1136	1024
	experimental^32^	2328	2323	1118	992
As	rel	2168	2154	1016	937
	no SO	2168	2154	1017	937
	nrel	2183	2171	1016	930
	experimental^32^	2123	2116	1003	906
Sb	rel	1933	1926	844	812
	no SO	1933	1926	842	809
	nrel	1961	1958	842	803
	experimental^34^	1894	1891	831	782
Bi	rel	1768	1766	764	750
	no SO	1796	1788	773	760
	nrel	1855	1852	765	742
	experimental^34^	1734	1733	751	726

a B3LYP functional, aug-cc-pVTZ (on H) + dyall.v3z (on X) basis set.

b No symmetry has been used, so frequencies of degenerate vibrations vary (by at most 2 cm^–1^). Arithmetic averages are given.

c Fundamental vibrational frequencies are reported for the experimental data. For the calculated results, the following notation is used: rel, relativistic; nrel, nonrelativistic; no SO, no spin–orbit coupling.

**Table 5:** Vibrational Frequencies for XH_4_: Comparison of Results Calculated with Relativistic and Nonrelativistic DFT Methods^a^

X	method^c^	ω_1_ [cm^–1^] (A_1_ symmetry, X–H symmetric stretch	ω_2_ [cm^–1^] (T_2_ symmetry, X–H asymmetric stretch)^b^	ω_3_ [cm^–1^] (E symmetry, H–X–H twist)^b^	ω_4_ [cm^–1^] (T_2_ symmetry, H–X–H scissor)^b^
C	rel	3135	3032	1555	1337
	no SO	3127	3025	1557	1339
	nrel	3127	3025	1556	1339
	experimental^32^	3019	2917	1534	1306
Si	rel	2237	2227	977	918
	no SO	2237	2227	977	918
	nrel	2238	2228	976	917
	experimental^32^	2191	2187	975	914
Ge	rel	2144	2136	934	827
	no SO	2143	2136	932	826
	nrel	2144	2139	925	823
	experimental^32^	2114	2106	931	819
Sn	rel	1929	1927	753	686
	no SO	1930	1927	752	684
	nrel	1930	1926	737	678
Pb	rel	1839	1827	686	609
	no SO	1847	1823	693	616
	nrel	1847	1839	664	609

a B3LYP functional, aug-cc-pVTZ (on H) + dyall.v3z (on X) basis set.

b No symmetry has been used, so frequencies of degenerate vibrations vary (by at most 2 cm^–1^). Arithmetic averages are given.

c Fundamental vibrational frequencies are reported for the experimental data. For the calculated results, the following notation is used: rel, relativistic; nrel, nonrelativistic; no SO, no spin–orbit coupling.

In addition, for all of the molecules, relativistic four-component calculations without spin–orbit effects have been performed (Table 4). These results show that in the case of XH_3_, all of the relativistic effects are in fact scalar relativistic effects, whereas in the case of H_2_X, spin–orbit effects play a crucial role. Spin–orbit effects constitute about 30% of the relativistic effects in the case of H_2_Te and as much as 70% in the case of H_2_Po.

### Comparison with Experimental Values

When comparing the results obtained with the experimental values, one should keep in mind that the diagonalization of the molecular Hessian gives us harmonic vibrational frequencies. Thus, anharmonicity is not taken into account, and this will lead to some difference between the results and the experimental values. In our case, the differences do not exceed 5%, in line with the expected magnitude of anharmonic corrections.^35^

### Vibrational Frequencies for HC≡CPbH_3_

To illustrate the usefulness of the presented method and to study the effects of relativity on vibrational frequencies for a more complex system where only some of the modes involve the heavy atom, we have calculated the vibrational frequencies for the acetylene derivative HC≡CPbH_3_ with both relativistic and nonrelativistic approaches. The motivation for choosing this particular system was our previous work,^15^ where we showed that for this molecule the relativistic effects on the derivatives of the indirect spin–spin coupling constants with respect to molecular geometry parameters tend to be more pronounced than the effects on the coupling constants themselves. The ZPV corrections calculated at the nonrelativistic level are therefore not necessarily reliable. The uncontracted aug-cc-pVDZ basis set^17^ was used on the hydrogen and carbon atoms and Dyall’s uncontracted triple-ζ basis set (dyall.v3z)^18−20^ on the lead atom have been applied together with the B3LYP exchange–correlation functional.^21−24^ The results are collected in Table 6.

**Table 6:** Vibrational Frequencies for HC≡CPbH_3_: Comparison of Results Calculated with Relativistic and Nonrelativistic DFT Methods^a^

mode	relativistic [cm^–1^]	nonrelativistic [cm^–1^]
C–H stretch (A_1_ symmetry)	3445	3440
C–C stretch (A_1_ symmetry)	2117	2116
Pb–H asymmetric stretch (E symmetry)^b^	1857	1840
Pb–H symmetric stretch (A_1_ symmetry)	1846	1846
C–C–H bend (A_2_ symmetry)^b^	708	702
H–Pb–H wag (A_1_ symmetry)	621	613
H–Pb–H scissor (E symmetry)^b^	641	597
H–C–C–Pb wag (A_2_ symmetry)^b^	482	429
C–Pb stretch (A_1_ symmetry)	384	409
C–C–Pb bend (A_2_ symmetry)^b^	187	145

a B3LYP functional, aug-cc-pVTZ (on H and C) + dyall.v3z (on Pb) basis set.

b No symmetry has been used, so frequencies of degenerate vibrations vary (by at most 5 cm^–1^). Arithmetic averages are given.

In the case of the HC≡CPbH_3_ molecule, it seems that only vibrations that involve the Pb atom are significantly affected by relativity. There is almost no difference between the relativistic and nonrelativistic values of vibrational frequencies for C–H stretching, C–C stretching, and C–C–H bending. This finding may be useful for future calculations of vibrational effects on molecular properties for large molecules, since it may allow for relativity to be taken into account only for selected localized modes. Similar findings were previously reported by Berger et al.^36^ In addition to this, the relativistic effects are much more pronounced for deformation modes than for stretching modes. The difference between the vibrational frequencies calculated with relativistic and nonrelativistic methods for the C–C–Pb bend exceeds 20% of the value, whereas for the C–Pb stretch it is only little more than 5%.

## Conclusions

We have presented a numerical method for calculating the molecular Hessian and harmonic vibrational frequencies with relativistic four-component DFT. Test calculations have been performed for hydrides of elements from groups 14, 15, and 16. We have achieved good agreement with an analytical nonrelativistic DFT method.

Relativistic effects become significant primarily for the hydrides containing atoms from the fifth and sixth rows of the periodic table and are much more pronounced for the vibrational modes with higher frequencies. Spin–orbit effects constitute a very small fraction of the relativistic effects on the whole, with the exception of H_2_Te and H_2_Po. Additional calculations for HC≡CPbH_3_ show that only the frequencies of the modes with large contributions from Pb displacements are significantly affected by relativity.

This work is considered a stepping stone towards the development of a four-component relativistic numerical method for calculating ZPV corrections to NMR parameters (spin–spin coupling constants and shielding constants).

## Supporting Information Available

Vibrational frequencies for H_2_O and H_2_Po obtained with different step lengths and different optimized geometries (PDF)
