# Supplementary material for: Relativistic Four-Component DFT Calculations of Vibrational Frequencies
Source: J Phys Chem A. 2021 Nov 29;125(48):10315–20. doi: 10.1021/acs.jpca.1c07398 (PMC8667032; doi:10.1021/acs.jpca.1c07398)
Supplement: Supplementary file 1 — jp1c07398_si_001.pdf [file jp1c07398_si_001.pdf]

Relativistic Four-component DFT Calculations of Vibrational Frequencies

Katarzyna Jakubowska,[†] Magdalena Pecul,[*,‡] and Kenneth Ruud[¶]

† *Faculty of Chemistry, University of Warsaw, Warsaw, Poland*

‡ *Faculty of Chemistry, University of Warsaw, 02-093 Warsaw, Poland*

¶*Hylleraas Centre for Quantum Molecular Sciences, Department of Chemistry, UiT – The Arctic University of Norway, N-9019 Tromsø, Norway*

E-mail: mpecul@chem.uw.edu.pl

Supporting Information

Vibrational frequencies for H_2O and H_2Po obtained with different step lengths (h).

Table S1: Vibrational frequencies for H_2O obtained with different step lengths (h).

h [Å]	0.05	0.01	0.005	0.001	0.0005	0.0001
rel	3941	3919	3918	3918	3918	3906
	3836	3816	3815	3815	3815	3802
	1621	1623	1623	1623	1623	1596
nrel	3943	3921	3920	3920	3919	3908
	3838	3818	3818	3817	3817	3805
	1621	1623	1623	1623	1622	1595

[a] 4-component DKS hamiltonian, basis set: aug-cc-pVTZ (on H) + dyall.v3z (on O), functional: B3LYP

Table S2: Vibrational frequencies for H_2Po obtained with different step lengths (h).

h [Å]	0.1	0.05	0.01	0.005	0.001	0.0005	0.0001
rel	1858	1848	1845	1845	1846	1847	1894
	1841	1831	1828	1828	1829	1830	1842
	773	775	775	775	777	776	807
							693
nrel	2045	2035	2032	2032	2030	2029	2022
	2044	2035	2031	2031	2029	2027	1928
	805	806	806	806	800	796	708

[a] 4-component DKS hamiltonian, basis set: aug-cc-pVTZ (on H) + dyall.v3z (on Po), functional: B3LYP

Optimized Geometries

All geometry optimization calculations have been carried out with the DIRAC program. The uncontracted aug-cc-pVDZ basis set on the hydrogen atoms and the uncontracted triple-ζ Dyall's basis set17–19 (dyall.v3z) on all the other atoms have been applied together with the B3LYP exchange-correlation functional. The xyz coordinates are given in Angstrom.

H_2O - rel

H	0.9613133760	0.0000000000	0.0052918765
H	-0.2435436859	0.0000000000	-0.9299677918
O	0.0009803099	0.0000000000	-0.0012640847

H_2O - nonrel

H	0.9621531629	0.0000000000	0.0055985260
H	-0.2440490289	0.0000000000	-0.9307052822
O	0.0006458659	0.0000000000	-0.0008332438

H_2O - no-so

H	0.9619978969	0.0000000000	0.0052781095
H	-0.2437001394	0.0000000000	-0.9306344259
O	0.0004522426	0.0000000000	-0.0005836837

H_2S - rel

H	1.1973804691	0.0000000000	0.0421453195
H	-0.3377929144	0.0000000000	-1.1495193477
S	-0.1408375547	0.0000000000	0.1814340282

H_2S - nonrel

H	1.1977547409	0.0000000000	0.0427881492
H	-0.3385084883	0.0000000000	-1.1497225287
S	-0.1404962525	0.0000000000	0.1809943795

H_2S - no-so

H	1.1973937353	0.0000000000	0.0421463232
H	-0.3377970656	0.0000000000	-1.1495314200
S	-0.1408466697	0.0000000000	0.1814450969

H_2Se - rel

H	1.2781237820	0.0000000000	0.0628855527
H	-0.3779103141	0.0000000000	-1.2225960196
Se	-0.1814634679	0.0000000000	0.2337704669

H_2Se - nonrel

H	1.2805785698	0.0000000000	0.0655273421
H	-0.3810783845	0.0000000000	-1.2243189485
Se	-0.1807501853	0.0000000000	0.2328516064

H_2Se - no-so

H	1.2777651166	0.0000000000	0.0626179152
H	-0.3775620692	0.0000000000	-1.2223148490
Se	-0.1814530474	0.0000000000	0.2337569338

H_2Te - rel

H	1.4139170141	0.0000000000	0.1070368483
H	-0.4543602670	0.0000000000	-1.3431963489
Te	-0.2408067471	0.0000000000	0.3102195006

H_2Te - nonrel

H	1.4196308146	0.0000000000	0.1128000416
H	-0.4613604131	0.0000000000	-1.3473020230
Te	-0.2395204015	0.0000000000	0.3085619815

H_2Te - no-so

H	1.4128827724	0.0000000000	0.1070083310
H	-0.4540768014	0.0000000000	-1.3422039688
Te	-0.2400559710	0.0000000000	0.3092556378

H_2Po - rel

H	1.4819462192	0.0000000000	0.1250917157
H	-0.4887263171	0.0000000000	-1.4046357492
Po	-0.2744699021	0.0000000000	0.3536040336

H_2Po - nonrel

H	1.4859317719	0.0000000000	0.1352246061
H	-0.4995289609	0.0000000000	-1.4059673977
Po	-0.2676528111	0.0000000000	0.3448027917

H_2Po - no-so

H	1.4672314632	0.0000000000	0.1224053243
H	-0.4824727192	0.0000000000	-1.3910332316
Po	-0.2660087439	0.0000000000	0.3426879073

NH_3 - rel

H	0.2689263565	0.6559375500	0.6865885359
H	0.7845316060	-0.4553946229	-0.3888170611
H	-0.5554421872	0.4193698339	-0.6997226045
N	-0.0423157753	-0.0527127610	0.0341511296

NH_3 - nonrel

H	0.2690999909	0.6564343159	0.6872422253
H	0.7851574718	-0.4558726549	-0.3891068237
H	-0.5559933506	0.4196593049	-0.7002861811
N	-0.0425641121	-0.0530209658	0.0343507795

NH_3 - no-so

H	0.2692222072	0.6564289146	0.6866981882
H	0.7850252581	-0.4553305513	-0.3891217333
H	-0.5554638294	0.4197698463	-0.7001472470
N	-0.0430836358	-0.0536682096	0.0347707922

PH_3 - rel

H	0.3522882298	0.8463985907	0.8631579655
H	1.0070480256	-0.5648499152	-0.5024551079
H	-0.6945485886	0.5459919306	-0.8972569413
P	-0.2090876667	-0.2603406061	0.1687540837

PH_3 - nonrel

H	0.3521795690	0.8465400113	0.8640247231
H	1.0073838528	-0.5656664767	-0.5025152739
H	-0.6953681196	0.5459295978	-0.8975853614
P	-0.2084953023	-0.2596031324	0.1682759121

PH_3 - no-so

H	0.3522822459	0.8463869083	0.8631500513
H	1.0070344934	-0.5648459935	-0.5024479916
H	-0.6945428259	0.5459834742	-0.8972455350
P	-0.2090739134	-0.2603243889	0.1687434753

AsH_3 - rel

H	0.3709463449	0.8930541379	0.9139797468
H	1.0633029099	-0.5992300344	-0.5300476568
H	-0.7359863852	0.5753943153	-0.9475088950
As	-0.2425628695	-0.3020184188	0.1957768050

AsH_3 - nonrel

H	0.3708736857	0.8946264584	0.9187193502
H	1.0659014458	-0.6034140987	-0.5308781522
H	-0.7403303691	0.5757420013	-0.9499507284
As	-0.2407447624	-0.2997543610	0.1943095304

AsH_3 - no-so

H	0.3709397976	0.8931534340	0.9142846347
H	1.0634677620	-0.5995000981	-0.5301006962

H	-0.7362646708	0.5754138198	-0.9476631043
As	-0.2424428888	-0.3018671557	0.1956791658

SbH_3 - rel

H	0.4042547589	0.9876606199	1.0365246093
H	1.1818910252	-0.6884297271	-0.5853618359
H	-0.8389844346	0.6308670731	-1.0542156139
Sb	-0.2914613495	-0.3628979659	0.2352528405

SbH_3 - nonrel

H	0.4045448841	0.9921864950	1.0480893152
H	1.1889060127	-0.6983910201	-0.5878130742
H	-0.8494801884	0.6323190542	-1.0607455025
Sb	-0.2882707084	-0.3589145291	0.2326692615

SbH_3 - no-so

H	0.4042801616	0.9875957224	1.0362641879
H	1.1817814571	-0.6881924504	-0.5853309693
H	-0.8387487759	0.6308725035	1.0541093956
Sb	-0.2916128428	-0.3630757755	0.2353761769

BiH_3 - rel

H	0.4183192084	1.0244482471	1.0795092004
H	1.2267607849	-0.7183283455	-0.6070020535
H	-0.8743754082	0.6533515478	-1.0945250915

Bi	-0.3150045851	-0.3922714494	0.2542179446

BiH_3 - nonrel

H	0.4203548129	1.0378388567	1.1082965304
H	1.2463970726	-0.7425304575	-0.6144814694
H	-0.9002371319	0.6588674701	-1.1125046480
Bi	-0.3108147536	-0.3869758693	0.2508895871

BiH_3 - no-so

H	0.4183273800	1.0234020981	1.0763929419
H	1.2251305286	-0.7156635576	-0.6064667377
H	-0.8715796598	0.6531485833	-1.0929085532
Bi	-0.3161782487	-0.3936871239	0.2551823490

CH_4 - rel

H	0.5290456846	0.1610914326	0.9363179348
H	0.2052132398	0.8243849488	-0.6788912803
H	0.3346592668	-0.9318368913	-0.4498076586
H	-1.0690087892	-0.0537287163	0.1922011265
C	-0.0000094021	-0.0000107738	-0.0000201225

CH_4 - nonrel

H	0.5295313847	0.1612517088	0.9371711176
H	0.2054201616	0.8251594756	-0.6795051003
H	0.3349768055	-0.9327110957	-0.4502224335

H	-1.0700089208	-0.0537806251	0.1923951063
C	-0.0000194310	-0.0000194637	-0.0000386901

CH_4 - no-so

H	0.5294728410	0.1612334128	0.9370648840
H	0.2053969979	0.8250660711	-0.6794279964
H	0.3349397169	-0.9326056227	-0.4501713715
H	-1.0698896892	-0.0537742429	0.1923728420
C	-0.0000198666	-0.0000196183	-0.0000383582

SiH_4 - rel

H	0.7212918080	0.2196372267	1.2765594029
H	0.2797949993	1.1239518614	-0.9255611272
H	0.4562786617	-1.2704346765	-0.6132395661
H	-1.4574551669	-0.0732430177	0.2620639188
Si	-0.0000103021	-0.0000113940	-0.0000226284

SiH_4 - nonrel

H	0.7215406224	0.2197122929	1.2770006537
H	0.2798906329	1.1243391193	-0.9258814092
H	0.4564355832	-1.2708723339	-0.6134511604
H	-1.4579571045	-0.0732682243	0.2621536436
Si	-0.0000097340	-0.0000108540	-0.0000217277

SiH_4 - no-so

H	0.7212835691	0.2196342656	1.2765461510
H	0.2797894419	1.1239364933	-0.9255508827
H	0.4562728888	-1.2704191431	-0.6132336263
H	-1.4574367443	-0.0732411493	0.2620591321
Si	-0.0000091554	-0.0000104665	-0.0000207741

GeH_4 - rel

H	0.7430784916	0.2262535813	1.3151262809
H	0.2882221762	1.1578733486	-0.9535272542
H	0.4700455361	-1.3087763335	-0.6317555339
H	-1.5014504094	-0.0754526447	0.2699575260
Ge	0.0000042055	0.0000020483	-0.0000010188

GeH_4 - nonrel

H	0.7462420643	0.2272167730	1.3207260028
H	0.2894491007	1.1628030606	-0.9575870742
H	0.4720466757	-1.3143482641	-0.6344451065
H	-1.5078420915	-0.0757737341	0.2711069500
Ge	0.0000042508	0.0000021646	-0.0000007721

GeH_4 - no-so

H	0.7431089129	0.2262627474	1.3151806054
H	0.2882335156	1.1579207014	-0.9535667580
H	0.4700645643	-1.3088299247	-0.6317816751
H	-1.5015114360	-0.0754556049	0.2699681785
Ge	0.0000044432	0.0000020808	-0.0000003508

SnH_4 - rel

H	0.8328683414	0.2535858909	1.4740439583
H	0.3230383437	1.2977683403	-1.0687428186
H	0.5268363788	-1.4668987516	-0.7080835835
H	-1.6828567169	-0.0845657720	0.3025700373
Sn	0.0000136531	0.0000102924	0.0000124065

SnH_4 - nonrel

H	0.8416794681	0.2562681626	1.4896397431
H	0.3264552364	1.3114986892	-1.0800502886
H	0.5324099720	-1.4824176861	-0.7155742341
H	-1.7006586463	-0.0854599542	0.3057710429
Sn	0.0000139699	0.0000107885	0.0000137367

SnH_4 - no-so

H	0.8329009742	0.2535950990	1.4741029983
H	0.3230505678	1.2978205773	-1.0687866934
H	0.5268569892	-1.4669569110	-0.7081114570
H	-1.6829224068	-0.0845690746	0.3025817712
Sn	0.0000138755	0.0000103092	0.0000133809

PbH_4 - rel

H	0.8529454128	0.2596969282	1.5095755218
H	0.3308197381	1.3290355640	-1.0944978318
H	0.5395283085	-1.5022394177	-0.7251459469

H	-1.7234082310	-0.0866038246	0.3098568297
Pb	0.0000147717	0.0000107502	0.0000114272

PbH_4 - nonrel

H	0.8823861447	0.2686621440	1.5616852962
H	0.3422429763	1.3749260848	-1.1322830792
H	0.5581584736	-1.5541089589	-0.7501790714
H	-1.7829035917	-0.0895920144	0.3205594256
Pb	0.0000159970	0.0000127445	0.0000174287

PbH_4 - no-so

H	0.8545177865	0.2601764098	1.5123613273
H	0.3314330320	1.3314990697	-1.0965225091
H	0.5405294377	-1.5050245262	-0.7264873852
H	-1.7265956486	-0.0867629475	0.3104334968
Pb	0.0000153924	0.0000119941	0.0000150701
